# The P2X7 receptor in mucosal adaptive immunity

**DOI:** 10.1007/s11302-023-09939-w

**Published:** 2023-04-17

**Authors:** Fabio Grassi, Rebecca Marino

**Affiliations:** Institute for Research in Biomedicine, Faculty of Biomedical Sciences, Università Della Svizzera Italiana, 6500 Bellinzona, Switzerland

**Keywords:** P2X7, Mucosal immunity, T cell, IgA, Gut, Microbiota

## Abstract

The P2X7 receptor (P2X7R) is a widely distributed cation channel activated by extracellular ATP (eATP) with exclusive peculiarities with respect to other P2XRs. In recent years, P2X7R has been shown to regulate the adaptive immune response by conditioning T cell signaling and activation as well as polarization, lineage stability, cell death, and function in tissues. Here we revise experimental observations in this field, with a focus on adaptive immunity at mucosal sites, particularly in the gut, where eATP is hypothesized to act in the reciprocal conditioning of the host immune system and commensal microbiota to promote mutualism. The importance of P2X7R activity in the intestine is consistent with the transcriptional upregulation of *P2xr7* gene by retinoic acid, a metabolite playing a key role in mucosal immunity. We emphasize the function of the eATP/P2X7R axis in controlling T follicular helper (Tfh) cell in the gut-associated lymphoid tissue (GALT) and, consequently, T-dependent secretory IgA (SIgA), with a focus on high-affinity SIgA-mediated protection from enteropathogens and shaping of a beneficial microbiota for the host.

## Introduction

Extracellular ATP is a well-known damage-associated molecular pattern (DAMP) that is released upon cell injury at inflamed and wounded sites. It is also released upon stimulation of pathogen recognition receptors (PRRs) by pathogen-associated molecular patterns (PAMPs) to promote the initiation of the innate immune response. The P2X7 receptor (P2X7R) is a nonselective cation channel activated by eATP that is widely distributed in both innate and adaptive immune system cells [1, 2]. Some exclusive peculiarities characterize P2X7R with respect to other P2XRs. First, the concentration of eATP needed to activate P2X7R is significantly higher than for other P2XRs [3]. Second, prolonged exposure to eATP causes the formation of a non-selective pore, which enables the passage of molecules of up to 900 Da and can lead to cell death. Various forms of P2X7R-mediated cell death have been described, among which pyroptosis is probably the best defined from a mechanistic point of view [4]. The formation of the large conductance pore mediated by P2X7R activation is dependent on the long carboxy-terminal tail of the receptor [5]*.* The structure of a rat P2X7R in both closed (apo state) and open (ATP-bound) states generated by cryo-electron microscopy (EM) has provided important insights on the receptor’s function; for example, the palmitoylation of the cytoplasmic C-cys anchor, which can prevent P2X7R desensitization, a general feature of other P2XR subtypes. Another structural element of P2X7R defined by cryo-EM has been the cytoplasmic ballast in the C-terminus, which endows the receptor with two peculiar features: a dinuclear zinc ion complex and a high-affinity guanosine nucleotide–binding site that could confer unique intracellular signaling properties [6]

The activation of the NLRP3 inflammasome by K^+^ efflux via stimulation of P2X7R is a paradigmatic example of the role purinergic signaling in immune cells can play in conditioning tissue homeostasis. Since eATP can have detrimental effects on physiological functions of the organism, its extracellular concentration is controlled by the activity of ubiquitous ectonucleoside triphosphate diphosphohydrolase 1 (NTPDase-1), which hydrolyze eATP into AMP, and ecto-5′-nucleotidase (NT5E), which generate immunosuppressive adenosine. The mucosal immune system provides the first line of protection at gastrointestinal, bronchopulmonary, and genitourinary barriers and is, therefore, exposed to a number of external stimuli, including eATP and others derived from microorganisms, which colonize these districts. A characterizing feature of mucosal sites is a layer of epithelium covered by mucus. The underlying connective tissue contains lymphocytes, dendritic cells (DCs), macrophages, and other cells, and secondary lymphoid structures, in which the local adaptive immune response is generated. The organized structures in which immune cells are concentrated are generally referred to as mucosal-associated lymphoid tissue (MALT). The first description of purinergic signaling in conditioning the adaptive immune system at a mucosal site was provided by Atarashi et al. by showing that eATP promoted the differentiation of intestinal Th17 cells and thereby could deteriorate intestinal inflammation [7].

## P2X7R signaling in lymphocytes

In 1989, Di Virgilio and coworkers provided the first evidence of T cell responsiveness to eATP via the yet-to-be-identified P2X7 receptor by showing increased plasma membrane depolarization and permeability in thymocytes by incubation with eATP. Interestingly, whereas cell death was eventually induced by hundreds of micromolar eATP in thymocytes, cytotoxic T lymphocytes (CTL) were resistant to eATP-induced plasma membrane depolarization and cell death, suggesting lack of expression of the eATP-responsive ionotropic receptor [8]. Subsequently, Filippini et al. showed that eATP hydrolysis during CTL activation inhibited the cytotoxic activity, suggesting purinergic signaling could synergize with TCR activation [9]. The identification of P2X7R paved the way to the demonstration of its contribution to mitogenesis in human T cells [10]. P2XR signaling was shown to promote the productive activation of mouse naïve CD4 T cells stimulated by cognate peptide/MHC antigen via an autocrine loop mediated by ATP released through pannexin 1 (PANX1) channels [11]. Because PANX1 hemichannels are found physically associated with P2X7R [12], ATP released through this pathway may reach a sufficiently high local concentration to induce P2X7R activation. Nevertheless, other P2XR isoforms, namely P2X1R and P2X4R, contributed to eATP-mediated signaling in naïve cells. P2XR stimulation constitutes a costimulatory signal, which exerts positive feedback on TCR-mediated mitogen-activated protein kinase (MAPK) activation. Analogous to mouse T cells, the eATP-P2XR axis promotes the productive activation of human T cells, where it contributes to Ca^2+^ influx and IL-2 production [13]. The function of P2X7R in promoting pro-inflammatory T cells activation was suggested in murine models of autoimmunity, where pharmacological antagonism of P2XRs dramatically ameliorated inflammatory tissue damage by promoting T cell anergy [11]. Moreover, ATP-P2X7 receptor signaling decreases the suppressive activity and favors the conversion of T regulatory (Treg) lymphocytes into T helper type-17 (Th17) cells [14].

In mouse T cells, the P2X7R can be activated also via poly-ADP ribosylation: NAD released during tissue injury and inflammation acts as a substrate for mono-ADP-ribosyltransferase (ART2) enzyme, which catalyzes the ADP-ribosylation of P2X7R on T cells. This activation promotes Ca^2+^ influx and shedding of CD62L, eventually leading to opening of the plasma membrane pore and DNA fragmentation, which configure the so-called NAD-induced cell death (NICD) [15]. NAD was specifically proposed as a regulator of Treg cell homeostasis through the ART2–P2X7 pathway by influencing survival, phenotype, and function [16]. In spite of the relevance of this mechanism in mice, premature stop codons inactivate *ART2* gene transcription in humans and chimpanzees, so ART2-mediated poly-ADP ribosylation of P2X7R is absent in these species [17]. An important outcome of P2X7R stimulation is the activation of membrane matrix metalloproteinases (MMPs). The respective enzymatic activity results in shedding of substrate molecules, including CD62L, CD27, and IL-6R, which can promote naïve T cell egress from secondary lymphoid organs (SLO) [18], condition the outcome of the T cell response [19] and signaling via IL-6R [20], respectively. In both mouse and human B cells, P2X7R-mediated MMP activation was shown to induce CD23 cleavage, thereby regulating the transendothelial migration of B cells [21].

## P2X7R in conditioning T cell development and function

Thymocytes are particularly sensitive to P2X7R-mediated cell death [22], probably as part of the thymocyte selection process. Different cytosolic Ca^2+^ responses elicited by P2X7R stimulation characterize thymocyte development. This observation led to hypothesize that loss of responsiveness to eATP via P2X7 could protect positively selected cells from cell death mediated by ATP released by neglected or negatively selected CD4^+^8^+^ double-positive (DP) thymocytes [23]. In fact, histone deacetylase (HDAC) 3 was shown to repress P2X7R signaling by binding to the *P2rx7* enhancer, thereby likely protecting positively selected DP cells from P2X7R-mediated cell death [24]. Other experiments suggest a more selective P2X7R function during T cell development in promoting γδ T cell lineage choice. In fact, ATP release and P2X7 signaling in γδTCR-expressing immature thymocytes act as an important costimulus for γδ T cell differentiation through the ERK-Egr-Id3 signaling pathway and also contribute to shaping the peripheral γδ T cell compartment. Conversely, lineage choice and differentiation to mature CD4 or CD8 αβ-expressing T cells seem not to be affected by P2X7R activity [25], made apart sensitivity to cell death mentioned above.

P2X7R activation inhibits the suppressive function and lineage stability of Foxp3-expressing Treg cells, which critically contribute to self-tolerance by the immune system [26]. P2X7R activity limits Foxp3 transcription and promotes Treg cell conversion to Th17 cell. The proinflammatory cytokine IL-6 induces ATP synthesis and ERK phosphorylation in Treg cell, thereby fostering autocrine signaling by P2X7R [14]. Treg cells are also exquisitely sensitive to P2X7R-mediated cell death [27]. The transcription factor HIF-1α that is induced by P2X7R stimulation in various cell types [28–30] has been shown to limit Treg versus Th17 cell differentiation by promoting the proteasomal degradation of Foxp3 [31]. An analogous P2X7R-mediated control of HIF-1α might condition Treg cell differentiation as well as IL-10-producing type-1 regulatory T (Tr1) cell. Accordingly, HIF-1α destabilizes the aryl hydrocarbon receptor (AHR), a transcription factor that drives the late phase of Tr1 cell differentiation [32].

T-cell dependent humoral immunity is conditioned by P2X7R activity. Tfh cell abundance is controlled by eATP/P2X7R-mediated pyroptosis; impairment of P2X7R responsiveness in Tfh cells unleashes B cell helper function and generation of antibodies reactive with self-antigens that characterize systemic lupus erythematosus (SLE) [33]. Interestingly, P2X7R activation in B cells inhibits BCR-dependent nuclear translocation of NF-ATc1, thereby limiting B cell activation [34]. The mechanisms of P2X7R activity in controlling B cell function in immunopathological conditions have been less extensively investigated than in T cells and need further investigation.

PRX7R was shown to promote mitochondrial homeostasis and metabolic function in differentiating memory CD8^+^ T cell via AMP-activated protein kinase [35] and generation of tissue-resident memory (Trm) cell by enhancing the sensitivity to TGF-β [36], thereby contributing to the establishment of long-lived T cells in mice. Nevertheless, P2X7R signaling in Trm cells induced cell death during infection and tissue damage. Since TCR triggering in Trm cell downregulates P2X7R, this observation suggested that sensing of eATP by P2X7R could favor antigen-specific over bystander Trm cells in the tissue niche [37]. Chronic exposure of CD8^+^ T cells to high eATP concentrations within solid tumors promotes cellular senescence via P2X7/ROS/p38MAPK/p21^Waf1/Cip1^ signaling, thus limiting anti-tumor cytotoxic activity [38]. Conversely, P2X7R stimulation of tissue-resident CD8^+^ T cells by eATP in the liver affected by nonalcoholic steatohepatitis (NASH) resulted in auto-aggressive killing of cells in an MHC-class-I-independent fashion [39]. Altogether, these data exemplify divergent context-dependent outcomes of P2X7R activity within different pathophysiological environments.

## Regulation of P2X7R activity in intestinal adaptive immunity by retinoic acid

**Fig. 1:**
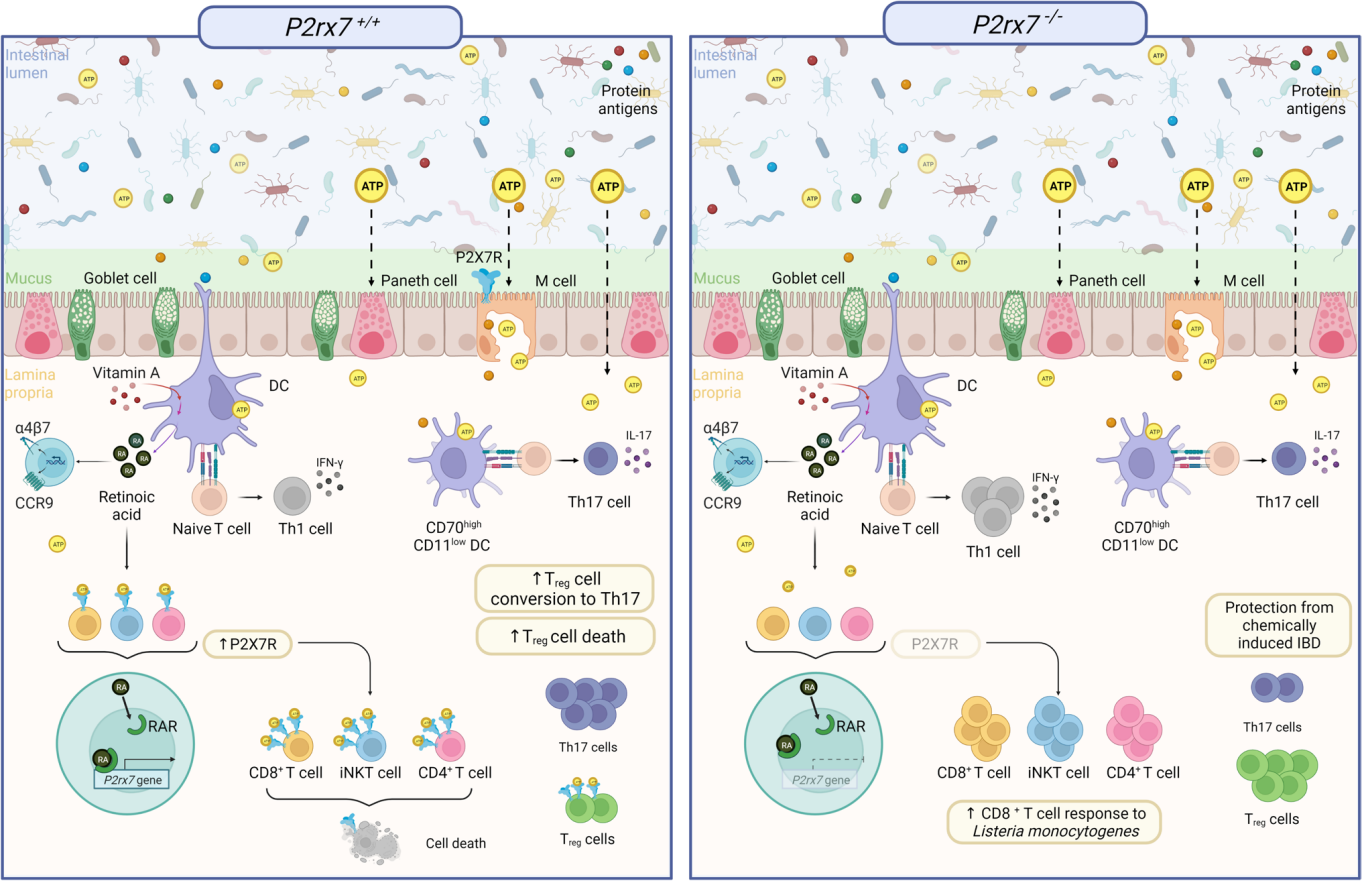
P2X7R mediated control of tissue resident effector T cells and Th17 polarization in the intestine. Luminal eATP is hypothesized to reach the intestinal lamina propria through Paneth cells, tight junctions or trancytosis by M cells. In *P2xr7*^*+/+*^ mice, RA promotes *P2rx7* transcription via binding of RAR complex to an intragenic enhancer region of P2rx7 in CD8, CD4 T and iNKT cell. P2rx7 upregulation renders these cell populations susceptible to P2X7R-mediated cell death, contributing to the regulation of T effector/memory and iNKT cells in the intestine. An eATP/P2XR axis can activate CD70 CD11 DCs, promoting Th17 polarization from naïve T cells. P2X7R signaling promotes Treg cell conversion to Th17 and also cell death, thereby further decreasing Treg cell number. In *P2rx7*^*-/-*^ mice, lack of P2X7R activity results in increased CD8 T cell response to *Listeria monocytogenes*. P2X7R deletion in Treg cell is associated with enhanced immunosuppression and protection from chemically induced IBD. Created with BioRender

The mucosal immune system consists of the GALT, including Peyer’s patches (PPs), mesenteric lymph nodes (MLNs), and isolated lymphoid follicles (ILFs), where the adaptive immune response is induced, and the intestinal epithelium and lamina propria (LP), where tissue-resident effector cells are located. Luminal antigens are sampled and captured by specialized epithelial microfold (M) cells and introduced into subepithelial lymphoid follicles in the PPs and ILFs. M cells are not antigen-presenting cells; rather, they transfer luminal particles and antigens to DCs for antigen presentation. They are also exploited by several invasive pathogens as portal for systemic infection. Interestingly, P2X7R was shown to be expressed at the apical site of M cells, suggesting luminal eATP could have a role in regulating the transcellular transport of intestinal content across the mucosal barrier into GALT and/or the expression of innate immune response genes by M cells via P2X7R signaling [40]. In addition to transcytosis via M cells, other mechanisms have been shown to allow sampling of the intestinal luminal content independently of organized lymphoid tissue. Extension of dendritic cell processes in between epithelial cells enables the capture of bacteria for direct uptake by dendritic cells [41], and goblet cells in the small intestine constitute passages for delivery of soluble antigens from the lumen to CD103^+^ DCs in the lamina propria [42]. When naïve B or T cells are activated in GALT, they are exposed to retinoic acid (RA) produced by the DCs. This induces the expression of the chemokine receptor CCR9, which allows homing back to the small intestine by sensing locally produced chemokine (C–C motif) ligand 25 (CCL25), and the integrin α4β7, which binds to mucosal addressin cell adhesion molecule (MAdCAM)-1 on the intestinal endothelium (Fig. 1).

The relevance of eATP/P2X7R in modulating intestinal T cell immunity was originally hypothesized by Heiss et al., who showed that intraepithelial CD8^+^ T cells in the small intestine expressed high levels of P2X7R and were particularly sensitive to cell death induced by the P2X7R agonists benzoyl-ATP (BzATP) and NAD^+^. This phenomenon was dependent on P2X7R stimulation since it was not observed with intraepithelial CD8^+^ T cells isolated from *P2rx7*^*−/−*^ mice. Conversely, CD8^+^ T cells isolated from the spleen and liver expressed low levels of P2X7R and were relatively resistant to P2X7R-mediated cell death. Notably, exposure of CD8^+^ T cells from the spleen and peripheral LNs to RA but not TGF-β potently upregulated P2X7R and conferred sensitivity to extracellular nucleotides, suggesting that P2X7R regulation depended on the small intestinal location and/or priming in GALT. Consistent with functional relevance of this regulatory mechanism in tissue-specific T cell responsiveness, *P2rx7*^*−/−*^ mice showed enhanced intestinal CD8^+^ T cell responses to infection with *Listeria monocytogenes* as compared to the P2X7R-proficient counterpart [43] (Fig. 1). RA-induced P2X7R activity is important for contraction of intestinal CD4^+^ effector/memory T cells as well. RA receptor α was shown to bind and induce histone H3 acetylation on an intragenic enhancer region of the *P2rx7* gene, thereby upregulating *P2rx7* transcription and rendering CD4^+^ T cells susceptible to P2X7R-mediated cell death. In line with the idea that P2X7R activity was important in controlling the size of the effector/memory CD4^+^ T cell pool, IFN-γ secreting Th1 and IL-17 secreting Th17 cells were significantly increased in the small intestine of *P2rx7*^*−/−*^ mice at steady state, and the increase became more pronounced upon infection of mice with *Citrobacter rodentium.* Furthermore, P2X7R-mediated cell death restrained inflammatory intestinal Th1 and Th17 cells and suppressed colitis. Therefore, in addition to being a key factor in promoting T cell migration to the intestine, RA is important for limiting the expansion of tissue-resident effector T cells in the intestine and controlling the development of inflammatory conditions [44].

A similar regulation of P2X7R as in effector/memory T cells has been observed in intestinal invariant natural killer T (iNKT) cells, which are responsive to glycolipid antigens and important regulators of the adaptive immune response at mucosal sites [45]. Tissue-resident iNKT cells in the intestine expressed high levels of P2X7R, and this expression was dependent on RA. Accordingly, in vitamin A deficiency, iNKT cells did not express P2X7R and were resistant to P2X7R-mediated cell death, and in *P2rx7*^*−/−*^ mice, iNKT cells expressing effector cytokines were significantly increased. Therefore, P2X7R activity is important to prevent aberrant expansion of effector cytokine-producing iNKT cells and ensures also iNKT cell homeostasis in the intestine [46] (Fig. 1).

## P2XR in Th17 cell polarization

As mentioned above, eATP affects the differentiation of Th17 effector cells from naïve T cells in the LP of the colon through the activation of a unique subset of CD70^high^CD11c^low^ DCs. However, whether eATP and P2X7R signaling were involved in this function was not established [7] (Fig. 1). Interestingly, Yamamoto et al. have recently hypothesized that purinergic stimulation of this DC subset in the nasal lamina propria via P2XRs might promote cytotoxic T cell responses secondary to the induction of CD4^+^ T cell differentiation to Th17 cells. The use of the P2XR agonist αβ-methylene ATP (αβ-ATP) as mucosal adjuvant in a model of cancer vaccination enhanced the control of tumor growth by antigen-specific Th17 and cytotoxic T cells [47]. Albeit other P2XR subtypes were possibly involved in Th17 cell differentiation in this particular experimental model, several lines of evidence show the function of the eATP/P2X7R axis in conditioning APCs to T cell polarization in pathophysiological conditions, such as in graft versus host disease (GVHD) [48], anti-cancer immunity [49], psoriasis [50], and type-II collagen-induced arthritis [51]. Along another line, in infection by *Toxoplasma gondii* and *Trichinella spiralis*, P2X7R in epithelial cells has been shown to be important in the initiation of small intestinal inflammation through chemokine production and recruitment of DCs to the site of infection, thereby contributing to the development of the adaptive immune response [52]. The pleiotropy of P2X7R in different cells of the immune system should be considered when drawing a picture on the involvement of this “ubiquitous” receptor in adaptive immunity.

## P2X7R in controlling intestinal secretory IgA

**Fig. 2:**
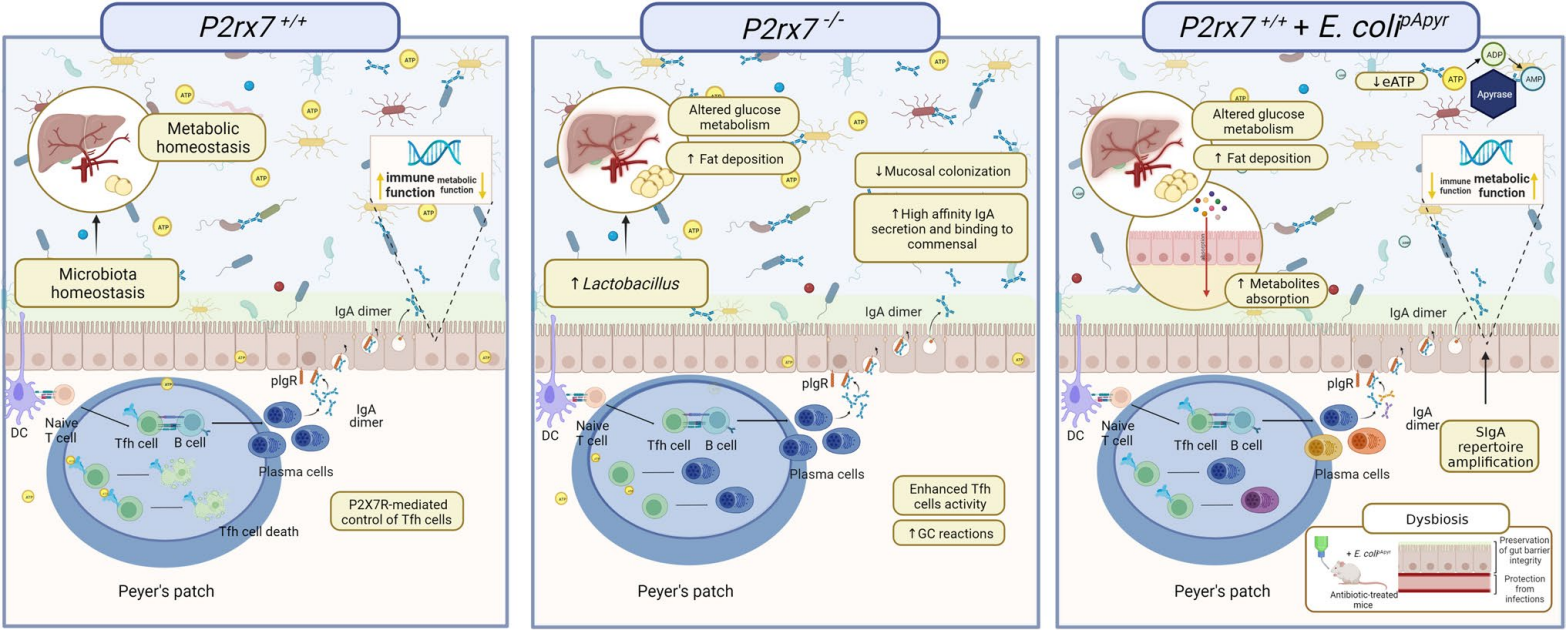
Control of Tfh cells abundance by P2X7R in the PPs of the small intestine and modulation of gut microbiota by SIgA. P2X7R limits Tfh cells abundance in the PPs, and consequently plasma cells generation and SIgA production. This mechanism ensures a physiological shaping of the microbiota, thus promoting metabolic homeostasis. In *P2rx7*^*-/-*^ mice, Tfh cell activity and GC reactions are enhanced. The resultant increase in SIgA affects microbiota composition, glucose metabolism and fat deposition. Reduction of intestinal eATP by apyrase enzymatic activity in mice monocolonized with *E. colip*^*Apyr*^ results in a more diversified SIgA repertoire and altered gene transcription in enterocytes by upregulation of genes involved in metabolic versus immune functions. Created with BioRender

Crucial in intestinal homeostasis and protection from enteropathogens is the generation of mucosal plasma blasts, which produce SIg. At steady state, plasma cells in the lamina propria secrete mainly IgA, which is bound by polymeric immunoglobulin receptor (pIgR) for transport across the epithelium and mucosal secretion. Isotype class switch to IgA in the gut can occur by a T-dependent or –independent mechanism. SIgA binding to intestinal resident microbiota and invading microbes plays a critical role in host microbiota mutualism and safeguard of the organism against infection, respectively. The intestinal homeostasis requires a delicate balance between activation of pro-inflammatory and immunosuppressive T cell subsets to ensure protection from pathogens and limit excessive immune activation. In this respect, SIgA regulates the composition, luminal confinement, and mucus colonization of the multitude of bacterial species that constitute the microbiota, thereby controlling the interaction of bacterial components with the local immune system. Tfh cells are instrumental in promoting SIgA affinity maturation in the germinal center, and this process in GALT is crucial for intestinal homeostasis and mucosal defense [53]. P2X7R expression in Tfh cells is potently downregulated at the transcriptional level by acute TCR stimulation. This mechanism enables expansion of antigen-specific cells and induction of eATP-mediated cell death of non-acutely stimulated cells, thereby ensuring physiological colonization of the intestine. *P2rx7*^−/−^ mice are characterized by Tfh cell resistance to cell death, enhanced germinal center reaction in PPs, high-affinity IgA secretion, and binding to commensals resulting in reduced bacterial mucosal colonization (Fig. 2). A consequence of this effect is the decreased absorption of bacterial components, such as LPS. Serum LPS acts as a “tonic” stimulator of B1 cells, and reduction of this steady state stimulation of the immune system in *P2rx7*^−/−^ mice results in reduction of serum IgM levels, exposing the mice to death following sublethal polymicrobial sepsis [54]. These observations suggest that eATP-mediated activation of P2X7R controls an important signaling pathway in shaping microbiota function for immune system homeostasis. Since eATP was able to induce cell death of Tfh cells isolated from human PPs, it seems plausible to hypothesize that abrogation of microbiota-derived eATP signaling could constitute a strategy to limit Tfh cell death and promote high-affinity mucosal IgA responses in humans.

This idea prompted testing whether high-affinity mucosal SIgA could be induced by immunizing mice with an appropriate vector in combination with apyrase (ATP-diphosphohydrolase that catalyzes the sequential hydrolysis of ATP to ADP and ADP to AMP releasing inorganic phosphate) to sustain Tfh cell’s activity. A live attenuated *Salmonella enterica* serovar Thypimurium carrying an expression plasmid for *Shigella flexneri’s* periplasmic apyrase (*S*. Tm^pApy^) that effectively hydrolyzed eATP in the ileum was generated as a proof-of-concept vaccine against infection by virulent *S*. Tm. Notably, this vaccine was significantly more effective in inducing high-affinity SIgA capable of clumping virulent *S*. Tm and neutralizing infection as well as systemic dissemination of the enteropathogen than control transformants not expressing apyrase (*S*. Tm^pBAD28^). This effect was dependent on the abrogation of P2X7R signaling in Tfh cell as *P2rx7*^*P2rx7-/-*^ mice did not show any improvement in the SigA response or protection from the infection by vaccination with *S*. Tm^pApy^ or *S*. Tm^pBAD28^ [55]. Enteric pathogens are a major health burden in both humans and animals. Moreover, antibiotic resistance often complicates the therapeutic approach to these infections underscoring the need for disease prophylaxis. These data indicate that apyrase can be used in mucosal vaccination protocols to elicit protective SIgA responses.

Tfh cell plays a role in host-microbiota interaction beyond protecting the intestinal mucosa by inducing affinity-matured SIgA. This cell type is poised to develop into memory T cell, and *P2rx7* gene upregulation is part of the transcriptional network characterizing this feature [56]. By chronically enhancing the production of species-specific SIgA, P2X7R-deficient Tfh cells promote altered intestinal colonization of individual taxa in *P2rx7*^*-/-*^ mice [57]. This effect has consequences for systemic metabolism, which critically depends on the composition of the intestinal microbiota [58]. In *P2rx7*^*-/-*^ mice, the modified microbiota dependent on enhanced Tfh cell activity and SIgA generation is responsible for altered glucose metabolism and abnormal sensitivity to high-fat diet. Therefore, bacteria-derived eATP in the gut is important for establishing a healthy relationship between microbiota, the adaptive immune response, and systemic homeostasis via P2X7R [57]. The SIgA-coated microbiota in *P2rx7*^*-/-*^ mice was characterized by the enrichment of bacteria typically residing in the small intestine, including *Lactobacillus, Enterococcus*, *and Enterobacteriaceae*. *Lactobacillus* was, by large, the most represented genus, and its richness correlated with body weight and abundance of Tfh cells in PPs. Moreover, *Lactobacillus* species enriched in the SIgA^+^ fraction of the microbiota from *P2rx7*^*-/-*^ mice could mimic the metabolic alterations observed in *P2rx7*^*-/-*^ mice once transplanted in microbiota-depleted mice, suggesting a causal relationship of this specific SIgA response in contributing to the metabolic phenotype of *P2rx7*^*−/−*^ mice (Fig. 2). Nevertheless, metagenomic analysis of the stools showed that deregulated SIgA in *P2rx7*^*−/−*^ mice conditioned intestinal microbial ecology beyond bacterial taxa that were selectively targeted by SIgA, further indicating the pervasive impact of the eATP/P2X7R axis on the physiological regulation of microbiota composition [59].

To understand whether P2X7R activity in intestinal Tfh cells had a function in specifically controlling the SIgA repertoire structure, we isolated plasma cells from the LP of mice monocolonized with *Escherichia coli* transformants either expressing apyrase (*E. coli*^pApyr^) or not (*E. coli*^pBAD28^) and performed high-throughput sequencing of Ig V_H_ regions. This analysis was particularly informative in revealing a more diversified SIgA repertoire in mice colonized with *E. coli*^pApyr^ and, therefore, devoid of intestinal eATP. Importantly, the enhanced breadth of the anti-*E. coli* SIgA response in mice colonized with *E. coli*^pApyr^ modified the topography of bacteria within the mucus layer and selectively conditioned the transcriptional activity in the epithelial component by favoring the upregulation of genes involved in metabolic functions in absorptive enterocytes versus genes involved in innate immunity. Accordingly, abrogation of luminal eATP affected metabolite absorption, body weight gain, glycemia, serum insulin, and fat deposition (Fig. 2). All these phenomena were dependent on the enhanced breadth of the SIgA response because the same analyses performed in IgA-deficient mice did not show any difference between mice monocolonized with *E. coli*^pApyr^ and *E. coli*^pBAD28^. These data unravel the importance of the complex interplay between microbiota and adaptive mucosal immunity in conditioning systemic metabolism and the function of eATP in mediating this reciprocal control via P2X7R [60]. SIgA amplification by apyrase could be exploited to beneficially affect gut microbial homeostasis during environmental distresses, such as in antibiotics-induced dysbiosis. In fact, apyrase could attenuate intestinal barrier impairment and glucose homeostasis perturbations in antibiotics-treated mice and promoted resistance to “opportunistic” enteric infection via SIgA [60] (Fig. 2).

## P2X7R in limiting Treg cell function in inflammatory bowel disease

Inflammatory bowel disease (IBD) regroups chronic intestinal inflammatory conditions characterized by severe gastrointestinal symptoms, Crohn’s disease (CD), and ulcerative colitis (UC) being the principal types. Various etiologies, including environmental and genetic factors, dysbiosis, impaired mucosal barrier, and intestinal immune system dysregulation, have been implicated in the pathogenesis of IBD. Familiar predisposition to develop either CD or UC has been recognized since long time [61], and different features leading to an abnormal response of the host immune system to intestinal microbial components have been hypothesized to contribute to the disease and its relapsing/remitting nature. Receptors for pathogen-associated molecular patterns (PAMPs) in innate immune system and epithelial cells as well as the balance between proinflammatory and immunosuppressive mechanisms of the adaptive immune system have been connected to IBD pathogenesis [62]. The contribution of P2X7R activity in many of these possible mechanisms has led to considering P2X7R antagonism as a therapeutic approach to IBD. However, in spite of encouraging preclinical results, a clinical trial with an orally available selective inhibitor of P2X7R did not show significant changes in inflammatory biomarkers, albeit significant amelioration of abdominal pain was observed [63]. A careful analysis of P2X7R expression in different cell types in the colon has shed light on some controversies on possible targets of P2X7R blockade in IBD [64], further suggesting cell-type specific functions conditioned by P2X7R should be carefully considered in developing therapies involving the receptor as a target.

In patients with CD, P2X7R expression was upregulated in the intestinal epithelium and lamina propria; apoptosis and proinflammatory cytokines were increased, whereas IL-10 levels were lower than in control samples [65]. In another study, lamina propria CD4^+^CD25^high^Foxp3^+^ Treg cells in the colon or ileum of CD showed increased apoptosis; notably, diminished apoptosis and increased number of Treg cells characterized the mucosa of subjects with disease resolution, suggesting a pathogenetic role for impaired immunosuppression by Treg cells in IBD [66]. The peculiar sensitivity of Treg cells to eATP/P2X7R-mediated cell death [67] could be important in this respect if one considers the local abundance of eATP due to bacterial colonization. Furthermore, P2X7R was shown to inhibit the suppressive activity of Treg cells and to promote Treg cell conversion to Th17. Adoptive transfer of a suboptimal number of P2X7R-deficient Treg cells in a model of IBD protected completely from the disease, and Treg cells did not convert to Th17 [14]. Experiments performed with *P2rx7*^*−/−*^ mice showed protection from chemically induced IBD; the resistance to inflammation was associated with increase in Treg cells in the lamina propria, prevention of Treg cell death, and increased IL-10 production [68]. Altogether, these experiments suggest P2X7R in Treg cells could be a possible target to control intestinal inflammation in IBD (Fig. 1).

A note should be added on the role of P2X7R in the relationship between IBD and intestinal tumorigenesis. In an azoxymethane (AOM)/dextran sodium sulfate (DSS) model of colitis-associated cancer, genetic and pharmacologic inactivation of P2X7R, while dampening DSS-induced inflammation, resulted in enhanced tumorigenesis [69]*.* This outcome due to P2X7R insufficiency is likely related to the modulation of the inflammatory tumor infiltrate by P2X7R. As expected, inflammasome activation and release of IL-1β were reduced in *P2rx7*^*−/−*^ mice treated with DSS; however, a dramatic increase of Treg cells in inflammatory and tumoral lesions was observed, suggesting loss of P2X7R activity could promote an adaptive immunosuppressive microenvironment and favor tumorigenesis. Moreover, TGF-β, known to promote Treg cell suppressive activity, was increased as was the infiltration of “tumor-associated neutrophils” [70] in colonic tumor tissue [69]. These findings indicate cautions should be used in considering P2X7R antagonism for dampening intestinal inflammation because of the possible tumor-promoting function of the treatment.

## P2X7R in the lung

Although lymphocytes are scarce at steady state in the airway and alveolar lumen, they are detected in the submucosa of bronchi. In infections and inflammatory conditions, they expand consistently, eventually leading to the formation of tertiary lymphoid structures defined as inducible bronchus-associated lymphoid tissue (iBALT). In asthmatic subjects and experimental models of asthma, eATP acutely accumulates in the airways upon challenge with the allergen. Broad spectrum pharmacological antagonism of P2 receptors improved all features of asthma, including eosinophilic airway inflammation, Th2 cytokine production, and bronchial hyper-reactivity. Moreover, eATP enhanced Th2 sensitization to inhaled antigen by recruiting and activating lung myeloid DCs that induced Th2 response in the mediastinal lymph nodes [71]. These data demonstrated that eATP-mediated P2 signaling plays a fundamental role in the pathogenesis of asthmatic airway inflammation. Pharmacological and genetic abrogation of P2X7R activity was associated with amelioration of acute and chronic asthma; the observation that P2X7R-deficient DC had a reduced capacity to induce the Th2 polarization and the upregulation of P2X7R in macrophages from bronchoalveolar fluid in patients with chronic asthma suggest P2X7R activity could condition the pro-asthmatic response of CD4 T cells in the pathogenesis of this respiratory condition [72]. Notably, in children at high risk of asthma because of viral illnesses and allergic sensitization in early life, attenuated P2X7 function was associated with lower asthma rates and sensitization to fewer aeroallergens [73]. Altogether, these data indicate that eATP/P2X7R axis might condition the outcome of the adaptive immune response in the respiratory tract.

Lung CD4 T cells were also induced by mucosal vaccination and mediated superior cross-protection against influenza virus variants than the immune response generated by systemic vaccination, highlighting their potential as a universal vaccine target [74]. P2RX7 expression characterizes a subset of lung CD4 T cells, defined T resident helper (Trh) cells, which differentiate within the organ and colocalize with B cells in iBALT following respiratory viral infection [75]. These cells appear important in local antibody production; however, whether and how P2RX7 modulates Trh cells function needs to be investigated.

Finally, eATP stimulation of P2X7R was shown to be important in promoting iNKT cell death during hyperoxic lung injury, a consequence of prolonged exposure to high-oxygen concentrations of critically ill patients. In fact, hyperoxia causes severe tissue damage in the lung, frequently leading to the death of the patient. Pulmonary iNKT cells play a crucial role in the development of this severe condition; by promoting iNKT cell death, P2X7R stimulation exerts a protective function against lung injury and significantly increases the survival in mice exposed to hyperoxia. Therefore, P2X7R in iNKT cells was proposed as a target for pharmacological intervention in hyperoxic lung injury [76].

## Conclusion

The function of P2X7R in the mammalian immune system has been intensely investigated in the last decades and attracted experts from different fields of immunology. From the relevance of extracellular ATP as DAMP and involvement of P2X7R in NLRP3 inflammasome formation and inflammatory caspase-1 activation in innate immune cells, more recent work has focused on P2X7R activity in adaptive immunity. In particular, the function of P2X7R stimulation in controlling T cell polarization, metabolism, memory formation, tissue residency, and susceptibility to cell death has been intensively investigated. The ubiquitous nature of ATP as signaling molecule in all kingdoms of life led us to hypothesize that it could act at the interface between mammalian hosts and microbiota by conditioning the mucosal-adaptive immune response via P2X7R and promoting host microbiota mutualism. Overall, the insights provided by the preclinical research targeting P2X7R within the adaptive immune system in different pathophysiological conditions should foster the interest of clinical researchers in this field and also lead to repurposing existing drugs for novel indications.

## Data Availability

The data discussed can be found in the original publications referenced in the text.

## References

[1] Junger WG (2011). Immune cell regulation by autocrine purinergic signalling. Nat Rev Immunol.

[2] Di Virgilio F, Dal Ben D, Sarti AC (2017). The P2X7 receptor in infection and inflammation. Immunity.

[3] Arulkumaran N, Unwin RJ, Tam FW (2011). A potential therapeutic role for P2X7 receptor (P2X7R) antagonists in the treatment of inflammatory diseases. Expert Opin Investig Drugs.

[4] Yang D, He Y, Muñoz-Planillo R (2015). Caspase-11 requires the pannexin-1 channel and the purinergic P2X7 pore to mediate pyroptosis and endotoxic shock. Immunity.

[5] Surprenant A, Rassendren F, Kawashima E (1996). The cytolytic P2Z receptor for extracellular ATP identified as a P2X receptor (P2X7). Science.

[6] McCarthy AE, Yoshioka C, Mansoor SE (2019). Full-length P2X7 structures reveal how palmitoylation prevents channel desensitization. Cell.

[7] Atarashi K, Nishimura J, Shima T (2008). ATP drives lamina propria TH17 cell differentiation. Nature.

[8] Di Virgilio F, Bronte V, Collavo D, Zanovello P (1989) Responses of mouse lymphocytes to extracellular adenosine 5’-triphosphate (ATP). Lymphocytes with cytotoxic activity are resistant to the permeabilizing effects of ATP. J Immunol 143:1955–1960 2789252

[9] Filippini A, Taffs RE, Sitkovsky MV (1990). Extracellular ATP in T-lymphocyte activation: possible role in effector functions. Proc Natl Acad Sci U S A.

[10] Baricordi OR, Ferrari D, Melchiorri L (1996). An ATP-activated channel is involved in mitogenic stimulation of human T lymphocytes. Blood.

[11] Schenk U, Westendorf AM, Radaelli E (2008). Purinergic control of T cell activation by ATP released through pannexin-1 hemichannels. Sci Signal.

[12] Pelegrin P, Surprenant A (2006). Pannexin-1 mediates large pore formation and interleukin-1β release by the ATP-gated P2X7 receptor. EMBO J.

[13] Yip L, Woehrle T, Corriden R (2009). Autocrine regulation of T-cell activation by ATP release and P2X7 receptors. FASEB J Off Publ Fed Am Soc Exp Biol.

[14] Schenk U, Frascoli M, Proietti M (2011). ATP inhibits the generation and function of regulatory T cells through the activation of purinergic P2X receptors. Sci Signal.

[15] Seman M, Adriouch S, Scheuplein F (2003). NAD-induced T cell death: ADP-ribosylation of cell surface proteins by ART2 activates the cytolytic P2X7 purinoceptor. Immunity.

[16] Hubert S, Rissiek B, Klages K (2010). Extracellular NAD+ shapes the Foxp3+ regulatory T cell compartment through the ART2-P2X7 pathway. J Exp Med.

[17] MacCallum RM, Martin AC, Thornton JM (1996). Antibody-antigen interactions: contact analysis and binding site topography. J Mol Biol.

[18] Sengstake S, Boneberg E-M, Illges H (2006). CD21 and CD62L shedding are both inducible via P2X7Rs. Int Immunol.

[19] Moon H, Na H-Y, Chong KH, Kim TJ (2006). P2X7 receptor-dependent ATP-induced shedding of CD27 in mouse lymphocytes. Immunol Lett.

[20] Garbers C, Jänner N, Chalaris A (2011). Species specificity of ADAM10 and ADAM17 proteins in interleukin-6 (IL-6) trans-signaling and novel role of ADAM10 in inducible IL-6 receptor shedding. J Biol Chem.

[21] Pupovac A, Geraghty NJ, Watson D, Sluyter R (2015). Activation of the P2X7 receptor induces the rapid shedding of CD23 from human and murine B cells. Immunol Cell Biol.

[22] Lépine S, Le Stunff H, Lakatos B (2006). ATP-induced apoptosis of thymocytes is mediated by activation of P2X7 receptor and involves de novo ceramide synthesis and mitochondria. Biochim Biophys Acta BBA - Mol Cell Biol Lipids.

[23] Ross PE, Ehring GR, Cahalan MD (1997). Dynamics of ATP-induced calcium signaling in single mouse thymocytes. J Cell Biol.

[24] Philips RL, McCue SA, Rajcula MJ (1950). Shapiro VS (2019) Cutting edge: HDAC3 protects double-positive thymocytes from P2X7 receptor-induced cell death. J Immunol Baltim Md.

[25] Frascoli M, Marcandalli J, Schenk U (1950). Grassi F (2012) Purinergic P2X7 receptor drives T cell lineage choice and shapes peripheral γδ cells. J Immunol Baltim Md.

[26] Hori S, Nomura T, Sakaguchi S (2003). Control of regulatory T cell development by the transcription factor Foxp3. Science.

[27] Taylor SRJ, Alexander DR, Cooper JC (1950). (2007) Regulatory T cells are resistant to apoptosis via TCR but not P2X7. J Immunol Baltim Md.

[28] Amoroso F, Falzoni S, Adinolfi E (2012). The P2X7 receptor is a key modulator of aerobic glycolysis. Cell Death Dis.

[29] Amoroso F, Capece M, Rotondo A (2015). The P2X7 receptor is a key modulator of the PI3K/GSK3β/VEGF signaling network: evidence in experimental neuroblastoma. Oncogene.

[30] Hirayama Y, Ikeda-Matsuo Y, Notomi S (2015). Astrocyte-mediated ischemic tolerance. J Neurosci Off J Soc Neurosci.

[31] Dang EV, Barbi J, Yang H-Y (2011). Control of T(H)17/T(reg) balance by hypoxia-inducible factor 1. Cell.

[32] Mascanfroni ID, Takenaka MC, Yeste A (2015). Metabolic control of type 1 regulatory T cell differentiation by AHR and HIF1-α. Nat Med.

[33] Faliti CE, Gualtierotti R, Rottoli E (2019). P2X7 receptor restrains pathogenic Tfh cell generation in systemic lupus erythematosus. J Exp Med.

[34] Pippel A, Beßler B, Klapperstück M, Markwardt F (2015). Inhibition of antigen receptor-dependent Ca(2+) signals and NF-AT activation by P2X7 receptors in human B lymphocytes. Cell Calcium.

[35] Borges da Silva H, Beura LK, Wang H (2018). The purinergic receptor P2RX7 directs metabolic fitness of long-lived memory CD8+ T cells. Nature.

[36] Borges da Silva H, Peng C, Wang H et al (2020) Sensing of ATP via the Purinergic Receptor P2RX7 Promotes CD8+ Trm Cell Generation by Enhancing Their Sensitivity to the Cytokine TGF-β. Immunity. 53:158–171.e6. 10.1016/j.immuni.2020.06.010 10.1016/j.immuni.2020.06.010 PMC8026201 32640257

[37] Stark R, Wesselink TH, Behr FM (2018). T RM maintenance is regulated by tissue damage via P2RX7. Sci Immunol.

[38] Romagnani A, Rottoli E, Mazza EMC (2020). P2X7 receptor activity limits accumulation of T cells within tumors. Cancer Res.

[39] Dudek M, Pfister D, Donakonda S (2021). Auto-aggressive CXCR6+ CD8 T cells cause liver immune pathology in NASH. Nature.

[40] Kim S-H, Lee H-Y, Jang Y-S (2015). Expression of the ATP-gated P2X7 receptor on M cells and its modulating role in the mucosal immune environment. Immune Netw.

[41] Rescigno M, Urbano M, Valzasina B (2001). Dendritic cells express tight junction proteins and penetrate gut epithelial monolayers to sample bacteria. Nat Immunol.

[42] McDole JR, Wheeler LW, McDonald KG (2012). Goblet cells deliver luminal antigen to CD103+ dendritic cells in the small intestine. Nature.

[43] Heiss K, Jänner N, Mähnss B (2008). High sensitivity of intestinal CD8+ T cells to nucleotides indicates P2X7 as a regulator for intestinal T cell responses1. J Immunol.

[44] Hashimoto-Hill S, Friesen L, Kim M, Kim CH (2017). Contraction of intestinal effector T cells by retinoic acid-induced purinergic receptor P2X7. Mucosal Immunol.

[45] Brailey PM, Lebrusant-Fernandez M, Barral P (2020). NKT cells and the regulation of intestinal immunity: a two-way street. FEBS J.

[46] Liu Q, Kim CH (2019). Control of tissue-resident invariant NKT cells by vitamin A metabolites and P2X7-mediated cell death. J Immunol.

[47] Yamamoto S, Matsuo K, Sakai S (2021). P2X receptor agonist enhances tumor-specific CTL responses through CD70+ DC-mediated Th17 induction. Int Immunol.

[48] Wilhelm K, Ganesan J, Müller T (2010). Graft-versus-host disease is enhanced by extracellular ATP activating P2X7R. Nat Med.

[49] Aymeric L, Apetoh L, Ghiringhelli F (2010). Tumor cell death and ATP release prime dendritic cells and efficient anticancer immunity. Cancer Res.

[50] Killeen ME, Ferris L, Kupetsky EA (1950). (2013) Signaling through purinergic receptors for ATP induces human cutaneous innate and adaptive Th17 responses: implications in the pathogenesis of psoriasis. J Immunol Baltim Md.

[51] Fan Z-D, Zhang Y-Y, Guo Y-H (2016). Involvement of P2X7 receptor signaling on regulating the differentiation of Th17 cells and type II collagen-induced arthritis in mice. Sci Rep.

[52] Huang S-W, Walker C, Pennock J et al (2017) P2X7 receptor-dependent tuning of gut epithelial responses to infection. Immunol Cell Biol 95:178–188. 10.1038/icb.2016.75 10.1038/icb.2016.75 PMC5181772 27559003

[53] Wei M, Shinkura R, Doi Y (2011). Mice carrying a knock-in mutation of Aicda resulting in a defect in somatic hypermutation have impaired gut homeostasis and compromised mucosal defense. Nat Immunol.

[54] Proietti M, Cornacchione V, RezzonicoJost T (2014). ATP-gated ionotropic P2X7 receptor controls follicular T helper cell numbers in Peyer’s patches to promote host-microbiota mutualism. Immunity.

[55] Proietti M, Perruzza L, Scribano D (2019). ATP released by intestinal bacteria limits the generation of protective IgA against enteropathogens. Nat Commun.

[56] Choi YS, Eto D, Yang JA (2013). Cutting edge: STAT1 is required for IL-6–mediated Bcl6 induction for early follicular helper cell differentiation. J Immunol.

[57] Perruzza L, Gargari G, Proietti M (2017). T follicular helper cells promote a beneficial gut ecosystem for host metabolic homeostasis by sensing microbiota-derived extracellular ATP. Cell Rep.

[58] Wang X, Zhang A, Miao J (2018). Gut microbiota as important modulator of metabolism in health and disease. RSC Adv.

[59] Perruzza L, Strati F, Gargari G (2019). Enrichment of intestinal lactobacillus by enhanced secretory IgA coating alters glucose homeostasis in P2rx7−/− mice. Sci Rep.

[60] Perruzza L, Strati F, Raneri M (2022). Apyrase-mediated amplification of secretory IgA promotes intestinal homeostasis. Cell Rep.

[61] Orholm M, Munkholm P, Langholz E (1991). Familial occurrence of inflammatory bowel disease. N Engl J Med.

[62] Baumgart DC, Carding SR (2007). Inflammatory bowel disease: cause and immunobiology. The Lancet.

[63] Eser A, Colombel J-F, Rutgeerts P (2015). Safety and efficacy of an oral inhibitor of the purinergic receptor P2X7 in adult patients with moderately to severely active Crohn’s disease: a randomized placebo-controlled, double-blind, phase IIa study. Inflamm Bowel Dis.

[64] Jooss T, Zhang J, Zimmer B (2023). Macrophages and glia are the dominant P2X7-expressing cell types in the gut nervous system - no evidence for a role of neuronal P2X7 receptors in colitis. Mucosal Immunol.

[65] Neves AR, Castelo-Branco MTL, Figliuolo VR (2014). Overexpression of ATP-activated P2X7 receptors in the intestinal mucosa is implicated in the pathogenesis of Crohn’s disease. Inflamm Bowel Dis.

[66] Veltkamp C, Anstaett M, Wahl K (2011). Apoptosis of regulatory T lymphocytes is increased in chronic inflammatory bowel disease and reversed by anti-TNFα treatment. Gut.

[67] Aswad F, Kawamura H, Dennert G (2005). High sensitivity of CD4+CD25+ regulatory T cells to extracellular metabolites nicotinamide adenine dinucleotide and ATP: a role for P2X7 receptors1. J Immunol.

[68] Figliuolo VR, Savio LEB, Safya H (2017). P2X7 receptor promotes intestinal inflammation in chemically induced colitis and triggers death of mucosal regulatory T cells. Biochim Biophys Acta BBA - Mol Basis Dis.

[69] Hofman P, Cherfils-Vicini J, Bazin M (2015). Genetic and pharmacological inactivation of the purinergic P2RX7 receptor dampens inflammation but increases tumor incidence in a mouse model of colitis-associated cancer. Cancer Res.

[70] Fridlender ZG, Sun J, Kim S (2009). Polarization of tumor-associated neutrophil phenotype by TGF-beta: “N1” versus “N2” TAN. Cancer Cell.

[71] Idzko M, Hammad H, van Nimwegen M et al (2007) Extracellular ATP triggers and maintains asthmatic airway inflammation by activating dendritic cells. Nat Med 13:13–919. 10.1038/nm1617 10.1038/nm1617 17632526

[72] Müller T, Vieira RP, Grimm M (2011). A potential role for P2X7R in allergic airway inflammation in mice and humans. Am J Respir Cell Mol Biol.

[73] Manthei DM, Jackson DJ, Evans MD (2012). Protection from asthma in a high-risk birth cohort by attenuated P2X7 function. J Allergy Clin Immunol.

[74] Zens KD, Chen JK, Farber DL (2016). Vaccine-generated lung tissue-resident memory T cells provide heterosubtypic protection to influenza infection. JCI Insight.

[75] Swarnalekha N, Schreiner D, Litzler LC (2021). T resident helper cells promote humoral responses in the lung. Sci Immunol.

[76] Nowak-Machen M, Schmelzle M, Hanidziar D (2013). Pulmonary natural killer T cells play an essential role in mediating hyperoxic acute lung injury. Am J Respir Cell Mol Biol.

